# Timing of Peripheral Blood Stem Cell Yield: Comparison of Alternative Methods with the Classic Method for CD34^+^ Cell Determination

**DOI:** 10.1155/2014/575368

**Published:** 2014-09-08

**Authors:** I. Fatorova, M. Blaha, M. Lanska, D. Vokurkova, V. Rezacova, P. Zak

**Affiliations:** ^1^4th Department of Internal Medicine-Hematology, Charles University, Medical Faculty and Teaching Hospital, Sokolska Street 581, 500 05 Hradec Kralove, Czech Republic; ^2^Institute of Immunology and Allergology, Charles University, Medical Faculty and Teaching Hospital, Sokolska Street 581, 500 05 Hradec Kralove, Czech Republic

## Abstract

Hematopoietic stem cells (HSCs), still represent a certain mystery in biology, have a unique property of dividing into equal cells and repopulating the hematopoietic tissue. This potential enables their use in transplantation treatments. The quality of the HSC grafts for transplantation is evaluated by flow cytometric determination of the CD34^+^ cells, which enables optimal timing of the first apheresis and the acquisition of maximal yield of the peripheral blood stem cells (PBSCs). To identify a more efficient method for evaluating CD34^+^ cells, we compared the following alternative methods with the reference method: hematopoietic progenitor cells (HPC) enumeration (using the Sysmex XE-2100 analyser), detection of CD133^+^ cells, and quantification of aldehyde dehydrogenase activity in the PBSCs. 266 aphereses (84 patients) were evaluated. In the preapheretic blood, the new methods produced data that were in agreement with the reference method. The ROC curves have shown that for the first-day apheresis target, the optimal predictive cut-off value was 0.032 cells/mL for the HPC method (sensitivity 73.4%, specificity 69.3%). HPC method exhibited a definite practical superiority as compared to other methods tested. HPC enumeration could serve as a supplementary method for the optimal timing of the first apheresis; it is simple, rapid, and cheap.

## 1. Introduction

Hematopoietic stem cells (HSCs) remain a mystery in biology as they can divide into completely equal daughter cells. This unique feature confers these cells the ability to completely repopulate the hematopoietic tissue. HSCs are therefore used in patient treatment of bone marrow transplantation. Successful peripheral blood stem cell (PBSC) transplantation depends on the infusion of an adequate number of hematopoietic stem cells to produce a rapid and durable hematological recovery. The number of CD34^+^ cells in the peripheral blood is widely used as a parameter for qualifying the engraftment potential of the PBSC concentrate. A minimum CD34^+^ cell count in the range of 0.010–0.020 cells/mL, depending on the number of leucocytes in the blood count, is recommended as the optimal cut-off value for initiating the harvest of PBSCs [1–3].

The level of CD34^+^ cells in the peripheral blood reaches a peak at different times, depending on the mobilisation regimen used. It is difficult to determine exactly when the peak levels of CD34^+^ cells are present in the peripheral circulation. Transplantation centres (including our centre) use the number of CD34^+^ cells in the peripheral blood for timing the initiation of apheresis procedures and this method is considered the “gold standard”. However, the clinical use of this test for the timing of apheresis is limited by logistical factors. The flow cytometry techniques currently used to measure CD34^+^ cells are relatively difficult to perform and are not very cheap. Additionally, some delay is usually required to obtain the results, which can complicate patient management and delay quick decisions. Although it has been accepted that the CD34^+^ cell count in the peripheral blood before leukapheresis is the best parameter for predicting CD34^+^ cell yield, other more easily measurable parameters are still used to guide the clinical practice of PBSC collection [4–6]. The white blood cell (WBC) count has been proposed as an easy prediction method; however, a number of studies, including ours, have argued that the correlation between the peripheral WBC count and the number of CD34^+^ cells is poor [7, 8]. However, identifying the most appropriate methods for the optimisation of PBSC aphereses for autologous or allogenic transplantation remains an active area of research.

Determination of the absolute number of CD34^+^ cells using flow cytometry is the reference method currently used at our centre. However, in our experience, this method of CD34^+^ determination is rather costly and time-consuming (exhibiting a standard response time of 1-2 hours).

Our team has been dealing with the problem of optimising the PBSC investigation both in the peripheral blood and in the collected product for many years. In particular, we have attempted to apply alternative methods and to introduce these methods into our guidelines for PBSC harvest. We used the XE-2100 analyser (Sysmex Corporation, Kobe, Japan) and the RF/DC method to determine the number of hematopoietic progenitor cells (HPCs). In addition, we quantified the number of CD133^+^ cells and the levels of the aldehyde dehydrogenase (ALDH) enzyme in the early leukocyte precursor cell population using flow cytometry and we compared the results of these three methods with the reference CD34^+^ method. The HPC determination method exhibited indisputable advantages in its simplicity, speed, and low cost. The application of this method is advantageous, especially for the detection of PBSCs in the peripheral blood [1]. However, the quantification of ALDH levels and CD133^+^ cell numbers also exhibit important advantages [4, 9]. Evidence of ALDH enzymatic activity in the apheresis product could provide precise and rapid information regarding the vitality of the graft before freezing [10, 11]. The presence of CD133^+^ cells, and the presence of double-positive CD34^+^/CD133^+^ cells in particular, could be used as an indication of the presence of highly immature progenitor cells in the apheresis product and could clarify the phenotype of this cell population [12].

In this work we investigated the HPC, ALDH, and CD133^+^ methods of PBSC quantification with the aim of clarifying both—if they correlate with the CD34^+^ standard flow cytometric determination and if they can provide more details on the classic CD34^+^ examination or if they even can replace the CD34^+^ determination.

## 2. Materials and Methods

The study was performed at the Department of Hematology of the University Hospital and Medical Faculty, Charles University, Hradec Králové, Czech Republic. The transplant program conformed to the Declaration of Helsinki and the Charles University Ethics Committee approved this study. All patients provided informed consent.

### 2.1. Patient Characteristics

The patients, 84 in total (47 males and 37 females; age range, 19–67 years; mean age, 53 years; median age, 58 years), were enrolled consecutively in the PBSC transplant program. In total, 266 PBSC apheresis procedures were performed and were evaluated over a three-year period (September 2007–March 2011). The characteristics of the patients and the laboratory results are shown in Tables 1, 2, and 3.

From a diagnostic point of view, multiple myelomas (MM, *n* = 49), Hodgkin and non-Hodgkin lymphomas (HL and NHL, *n* = 24), acute lymphoblastic leukaemia (ALL, *n* = 3), solid tumours (*n* = 2), and healthy donors (*n* = 6) were observed.

### 2.2. Mobilisation and PBSC Harvest

The patients received the appropriate high-dose cytostatic chemotherapy in accordance with their diagnoses [13]. The cytostatic treatment was followed by the administration of growth factors (G-CSF) at a dose of either 5 or 10 *μ*g/kg/day (5 *μ*g/kg/day was used for the lymphomas and ALL, and 10 *μ*g/kg/day was used for the MM). The healthy donors were administered only a growth factor (G-CSF) at a dose of 5 *μ*g/kg/day.

Complete blood counts were performed daily before the apheresis procedure. The cut-off value for initiating the harvest of PBSCs was 0.010 CD34^+^ cells per mL, provided the WBC count reached at least 1 × 10^6^ cells/mL. The harvest was not performed in one patient because of an insufficient response to the growth factor stimulation. The criterion for optimal PBSC collection was the target number of 5 × 10^6^ CD34^+^ cells/kg of patient's body weight. Once initiated, the leukapheresis was performed daily in an attempt to achieve this goal.

The apheresis was performed using the Cobe Spectra continuous flow blood cell separator (Terumo BCT, Lakewood, CO, USA; software version 4 MNC) or the Optia (Terumo BCT, Lakewood, CO, USA). The CD34^+^ determination was performed on the leukapheresis products before cryopreservation. The leukapheresis products were cryopreserved using 10% dimethyl sulphoxide in a controlled-rate freezing process. Venous access was established via either a peripheral vein or a central venous catheter and the anticoagulant solution ACD-A (Baxter, Munich, Germany) was infused at a ratio of 1 : 12–1 : 16 (depending on the thrombocyte count). The collection rate was maintained at 0.7–0.9 mL per minute. We attempted to use 3-4 total blood volumes for the wash procedure.

### 2.3. Enumeration of HPCs

**Figure 1:**
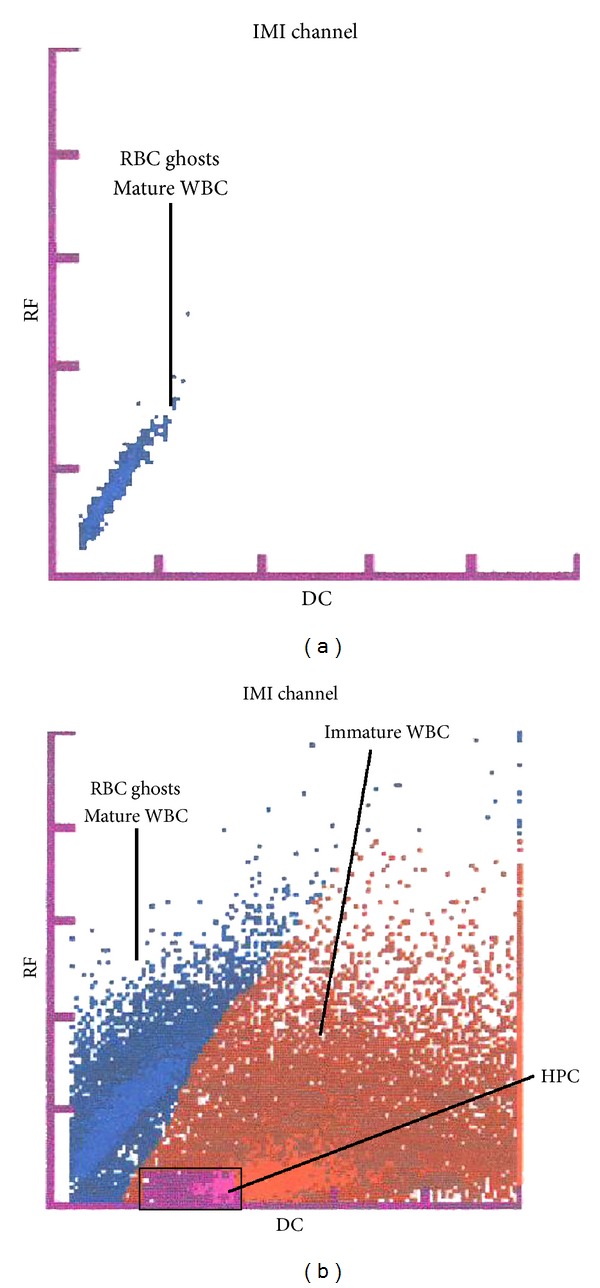
Determination of hematopoietic progenitor cells (HPC). HPC determination using the Sysmex XE-2100 analyzer on the immature myeloid information (IMI) channel for detection of immature hematopoietic cells. (a) A negative finding in which only areas with mature leukocytes (WBC) and traces/ghosts of erythrocytes (RBC) are visible. (b) A positive finding exhibiting an area of immature cells of the myeloid series (brown area) and an area of HPC presence (violet area). RF = radio frequency; DC = direct current.

The blood was collected in test tubes containing potassium (K3) ethylene diamine tetraacetic acid (K_3_EDTA). The enumeration of the HPCs was performed in 100 *μ*L of blood and additional blood count parameters were measured. The data was obtained approximately 15 minutes after the laboratory received the sample. The enumeration of the HPCs was performed using the Sysmex XE-2100 analyser (Sysmex Corporation, Kobe, Japan) in the immature myeloid information (IMI) channel using radio frequency (RF) and direct current (DC) methods to measure the cell size and density [1, 14]. In addition, there was a proprietary lytic reagent (Stromatolyser-IM) in the IMI channel. The Stromatolyser-IM reagent lyses the erythrocytes and mature leukocytes but does not lyse the immature myeloid cells that exhibit lower membrane lipid contents [15, 16]. In the IMI scattergram, the HPCs appeared in a distinct area of the blast region. The number of HPCs is presented as an absolute number and as a percentage of the WBC in the sample. The method is shown in Figure 1.

### 2.4. ALDH Determination

**Figure 2:**
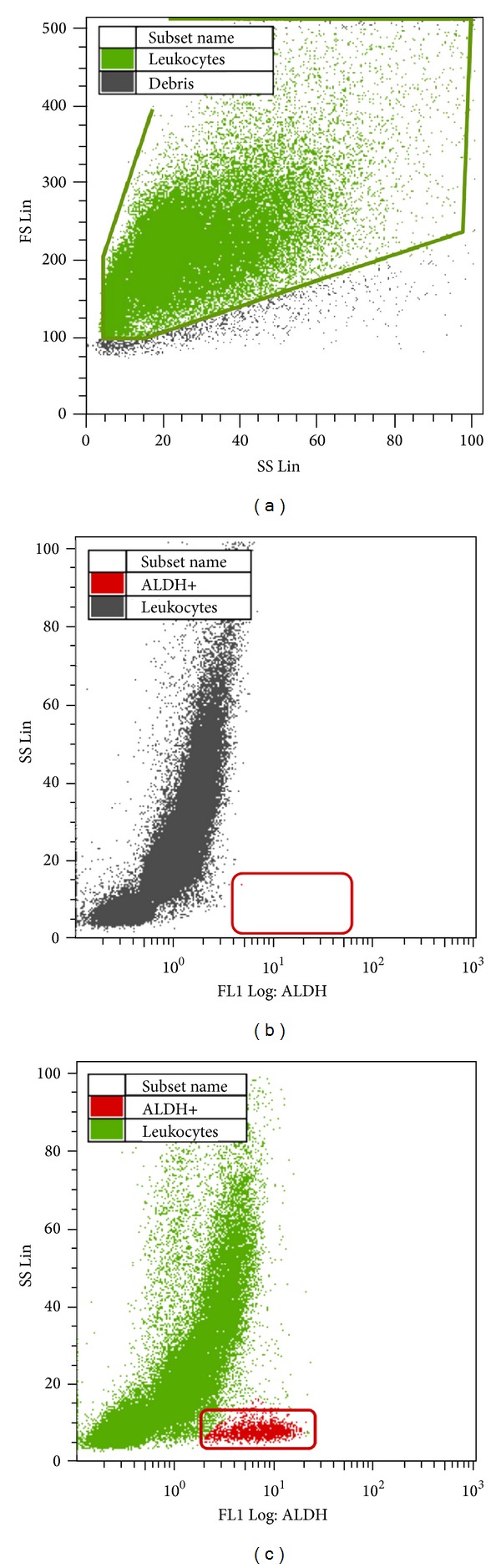
ALDH determination. Aldehyde dehydrogenase (ALDH) determination using flow cytometry. (a) Borders of the area of white blood cells (WBCs). (b) Negative control. (c) Evidence of ALDH-positive cells after staining using Aldefluor reagent (BAAA). SS Lin = side-scatter linear scale; FS Lin = forward-scatter linear scale; FL1 Log = fluorescent logarithmic scale.

The expression of the ALDH enzyme in the heparinised blood was assayed using the Aldefluor assay (Aldagen, Durham, CA, USA) on a Coulter Epics XL flow cytometer (Beckman Coulter, Brea, CA, USA) in accordance with the method described in Storms et al. [10, 17]. In this method, an alternative fluorescent substrate for ALDH, termed BODIPY aminoacetaldehyde (BAAA), was used to search for human hematopoietic progenitors that expressed ALDH. The method involves the lysis of erythrocytes as follows: 20 mL of ammonium chloride-based Aldecount lysis buffer (Aldagen, Durham, CA, USA) was added to 0.5 mL of blood and the sample was gently mixed and incubated at room temperature for 30 minutes. The sample was centrifuged at 250 ×g for 5 minutes and the supernatant was removed. The leukocyte fraction (1 × 10^6^ WBC/mL) was prepared by adding assay buffer (Aldagen, Durham, CA, USA) to the cell pellet. Next, 0.5 mL of the leukocyte fraction was incubated with 5 *μ*L of the fluorescent reagent BAAA, which is specific for the aldehyde dehydrogenase expressed in highly immature precursors of leukocytes, for 30–60 minutes at 37°C [10, 18]. After incubation, the mixture was centrifuged at 250 ×g for 5 minutes, the supernatant was removed, the cell pellet was resuspended in 0.5 mL of assay buffer (Aldagen, Durham, CA, USA), and the mixture was analysed immediately. The procedure for determining the presence of ALDH-positive cells is shown in Figure 2.

### 2.5. CD34^±^/CD133^±^ Determination

We determined the numbers of CD34^+^ and CD133^+^ cells in the heparinised blood using tricolour flow cytometry on a Coulter Epics XL analyser (Beckman Coulter, Brea, CA, USA). We incubated 100-*μ*L samples (the WBC count was adjusted to 1 × 10^6^ WBC/mL using PBS buffer, pH 7.2; Immunotech, Tampa, FL, USA) with the following antibodies: 5 *μ*L of anti-CD34 (FITC-labelled) (Immunotech, Tampa, FL, USA), 5 *μ*L of anti-CD45 (PC5) (Immunotech, Tampa, FL, USA), and 5 *μ*L of anti-CD133 (PE-labelled) (Miltenyi Biotec, Bergisch Gladbach, Germany) in 1 mL of lysing solution (VersaLyse, Immunotech, Tampa, FL, USA) for 15 minutes at room temperature. The mixture was centrifuged for 5 minutes at 150 ×g and the supernatant was removed. The cell pellet was washed three times in 3 mL of PBS buffer. After the last wash and centrifugation (5 minutes at 150 ×g) step, we resuspended the cell pellet in 1 mL of PBS buffer and the analysis was performed within 2 hours. The absolute number of labelled CD34^+^ cells was evaluated based on the ISHAGE guidelines (International Society of Hematotherapy and Graft Engineering protocol) described elsewhere [19, 20].

**Figure 3:**
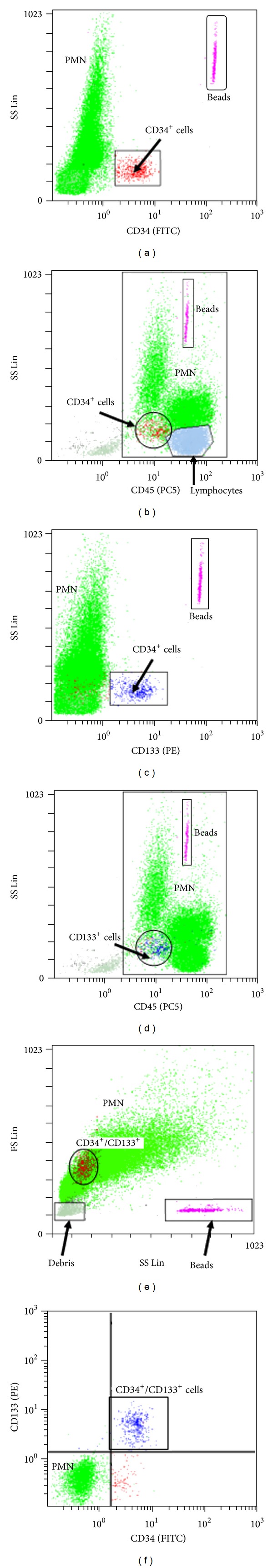
Determination of CD34^+^ and CD133^+^cells. Determination of CD34^+^ and CD133^+^ cells using flow cytometry (modified ISHAGE protocol). Tricolor flow cytometry CD34^+^ (FITC)/CD45^−^ (PC5)/CD133^+^ (PE). ((a), (b)) the population of CD34^+^ cells. ((c), (d)) the population of CD133^+^ cells. ((e), (f)) the population of CD34^+^/CD133^+^ cells. FITC = fluorescein isothiocyanate; PE = phycoerythrin; PC5 = phycoerythrin-cyanine5; FS Lin = forward-scatter linear scale; SS Lin = side-scatter linear scale.

We used a modified protocol enriched with the CD133 antibody. This antibody was conjugated with phycoerythrin (PE); therefore, we could not use the standard PE-labelled CD34 antibody and choose a suitable alternative, FITC-labelled anti-CD34. The FITC-labelled anti-CD34 monoclonal antibody (mAb) 581 specifically recognises class III and is suitable for use in the ISHAGE protocol. The protocol, including the identification of the double-positive population of CD34^+^/CD133^+^ cells, is shown in Figure 3.

### 2.6. Statistical Evaluation

We evaluated the descriptive statistics (median, mean, and range) for each of the methods in every group, before and after the PBSC collection, using the Excel Microsoft Office 2007 Standard program (Microsoft Corporation, Redmond, WA, USA). For the HPC method, we also examined the repeatability (i.e., the accuracy in the series) and linearity of the measurements in the samples from the apheresis products. We performed further statistical analyses using the MedCalc software program (MedCalc, Mariakerke, Belgium). After generating the receiver operating characteristic (ROC) curves, we evaluated the sensitivity, specificity, area under the curve (AUC), positive predictive value (PPV), and negative predictive value (NPV) for each test [21]. The AUC is a marker of the test quality (an AUC value of 1–0.97 is excellent, 0.97–0.92 is very good, 0.92–0.75 is good, and 0.75–0.50 represents an applicable test). After rejection of the normal distribution of the data (D'Agostini test; a *P*-value <0.05 was considered statistically significant), we used Spearman's nonparametric correlation coefficient (*R*) to correlate the data of the individual alternative methods with the reference method.

## 3. Results

The healthy donors underwent only 1 or 2 blood collections, producing a very good yield of CD34^+^ cells (3.41 × 10^6^–9.04 × 10^6^ CD34^+^ cells/kg). The patients underwent 3 consecutive collections on average (range 2–6) (Table 1). A low yield, even with repeated harvests (4–6), was obtained in 6 of the MM patients. For all samples, the laboratory results were acquired in parallel for the following methods: the absolute HPC count (using the Sysmex XE-2100 analyser), the absolute count of the CD34^+^ and CD133^+^ cells based on the modified ISHAGE guidelines (using a Coulter Epics XL flow cytometer), and the absolute number of ALDH-positive cells (using a Coulter Epics XL flow cytometer). The investigations were performed on the peripheral blood before the collection of the apheresis samples and on the apheresis product.

### 3.1. Descriptive Statistics: Linearity and Repeatability of the HPC Method

The results of the descriptive statistics are shown in Table 1. In addition, we demonstrated the linearity and repeatability of the measurements in samples of the apheresis product in which the leukocyte count was often found to exceed the degree of linearity guaranteed by the manufacturer of the Sysmex XE-2100 analyser (guaranteed for 0.00–100.0 × 10^6^ WBC/mL).

We verified the linearity by measuring the WBC and HPC amounts in 20 samples exhibiting extreme cellularity (WBC > 100.0 × 10^6^ cells/mL in the apheresis products). We analysed undiluted samples and samples diluted at ratios of 1 : 1, 1 : 2, 1 : 4, and 1 : 8 using the Cellpack diluent (Sysmex Corporation, Kobe, Japan). We generated a correlation coefficient for each sample and an average correlation coefficient for all 20 samples. The average correlation for the WBC was 0.998 (range 0.988–1) and was 0.964 (range 0.806–0.999) for the HPCs, and our results demonstrated a good linearity for the measurements of samples with high cellularity (i.e., WBC counts > 100.0 × 10^6^ cells/mL).

The repeatability of the method (i.e., the accuracy in the series) was verified by the repeated measurements of 20 samples from the apheresis product. We performed 5 consecutive measurements for each sample and determined the coefficient of variation (CV) for the WBC and HPC parameters of each sample. Subsequently, we calculated the average CV value for the WBCs and HPCs from all 20 samples. The average CV value for the WBCs was 0.9% (range 0.4–1.7%) and for the HPCs was 11.4% (range 2.3–20.5%), and these values were within the specifications provided by the manufacturer of the cell analyser.

### 3.2. Evaluation of ROC Curves

The sensitivity, specificity, PPV, NPV, and AUC were determined from the ROC curves. The confidence interval (CI) was 95%. For the evaluation, the values obtained by measuring the peripheral blood samples were used. Because the WBC values in a number of individuals did not reach 5 × 10^6^ cells/mL on the day of collection, the cut-off value for the CD34^+^ method was 0.020 cells/mL, which is consistent with the other published results.

For HPC and ALDH determinations, the sensitivity decreased and the specificity increased significantly with increasing numbers of positive cells in the peripheral blood. For the first-day apheresis target, the optimal predictive cut-off values were 0.032 cells/mL for the HPC method (sensitivity 73.4%, specificity 69.3%) and 0.033 cells/mL for the ALDH method (sensitivity 63.6%, specificity 84.9%). Both the HPC and ALDH tests exhibited a high quality level because the analyses were performed on samples with AUC values of 0.766 and 0.807 for the HPC and ALDH methods, respectively.

**Figure 4:**
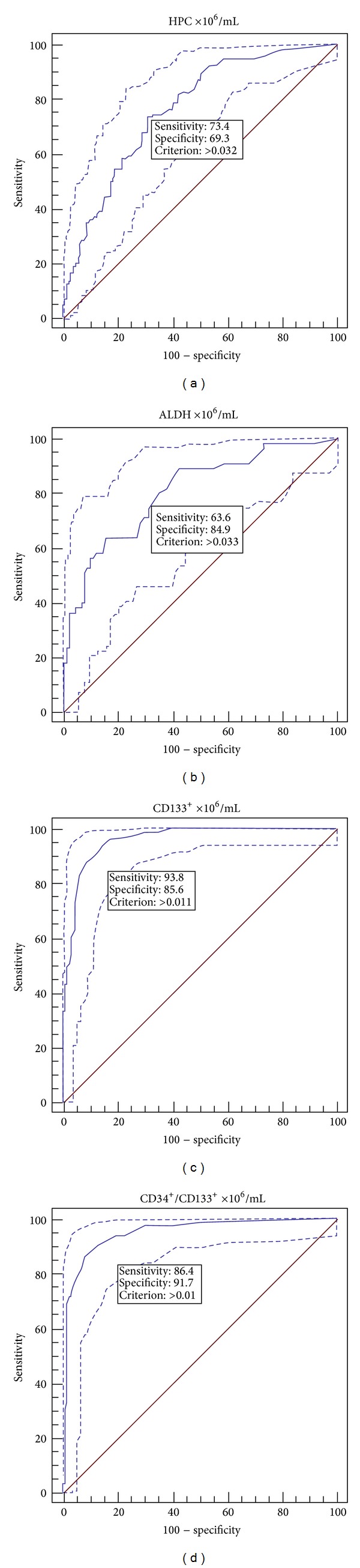
ROC curves. ROC curves for determination of method sensitivity and specificity at a cut-off value for CD34^+^ cells of 0.020 cells/mL (in peripheral blood). (a) HPC method (*n* = 264), (b) ALDH method (*n* = 145), (c) CD133^+^ method (*n* = 178), and (d) CD34^+^/CD133^+^ method (*n* = 178).

The specificity and sensitivity of the CD133^+^ and double-positive CD34^+^/CD133^+^ cell determinations were very good. The optimal predictive cut-off values of 0.011 cells/mL for the CD133^+^ count in the preapheresis peripheral blood were associated with high sensitivity and specificity values of 93.8% and 85.6%, respectively. The CD34^+^/CD133^+^ method predictive cut-off value was 0.010 cells/mL with a sensitivity and specificity of 86.4% and 91.7%, respectively. The CD133^+^ and CD34^+^/CD133^+^ methods were of a very high quality, exhibiting a high AUC value. For the CD133^+^ method, the AUC value was 0.959 and was 0.948 for the CD34^+^/CD133^+^ method (Figure 4).

### 3.3. Correlations

Spearman's correlation analysis of the preleukapheresis circulating HPCs, ALDH-positive cells and CD133^+^ and CD34^+^/CD133^+^ cells relative to the CD34^+^ cells demonstrated positive correlations in all cases at a 95% confidence interval (CI). For the HPC and ALDH methods, the correlation was strong (*R* = 0.604/0.721). The correlation was very strong for the CD133^+^ cell (*R* = 0.940) and CD34^+^/CD133^+^ cell (*R* = 0.933) methods.

**Figure 5:**
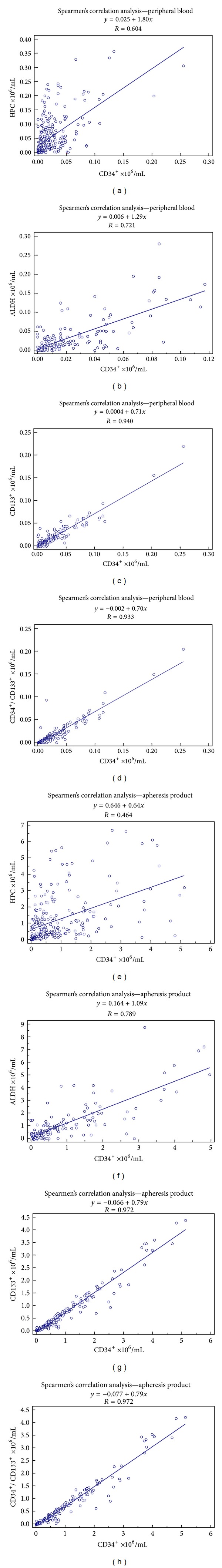
Spearman's nonparametric correlation graphs. Spearman's correlation analysis in the peripheral blood between CD34 method and (a) HPC method (*R* = 0.604, *n* = 264), (b) ALDH method (*R* = 0.721, *n* = 145), (c) CD133 method (*R* = 0.940, *n* = 178), and (d) CD34/CD133 method (*R* = 0.933, *n* = 178). Spearman's correlation analysis in the apheresis product between CD34 method and (e) HPC method (*R* = 0.464, *n* = 264), (f) ALDH method (*R* = 0.789, *n* = 142), (g) CD133 method (*R* = 0.972, *n* = 182), and (h) CD34/CD133 method (*R* = 0.972, *n* = 182).

The correlations between the CD34^+^ cell count (i.e., the reference cell count) in the apheresis product and the ALDH, CD133^+^ and CD34^+^/CD133^+^ methods were very strong at a 95% confidence interval (*R* = 0.789, 0.972, and 0.972, resp.). However, correlation analysis between the HPC and the reference CD34^+^ positive cell number demonstrated only a weak correlation in the apheresis product (*R* = 0.464). The results are shown in Figure 5.

## 4. Discussion 

In this study, we sought a precise, rapid, and economical alternative method for both the optimal timing of apheresis and the best prediction of a high quality graft (rich in PBSCs) for which the determination of the number of CD34^+^ cells using the ISHAGE protocol has been considered the “gold standard” [19, 20]. The four alternative methods investigated (determination of HPCs, ALDH, CD133^+^ cells, and CD34^+^/CD133^+^ cells) exhibited good or very good sensitivity, specificity, and accuracy (quality) compared with the samples from the preapheresis peripheral blood (Figures 4 and 5). The HPC determination method demonstrated very good linearity (*R* = 0.964) and repeatability (CV = 11.4%), even when the samples contained extremely high WBC numbers (up to 500 × 10^6^ WBC/mL). The CV of the leukocyte count (0.9%), a measure of repeatability, was within the range guaranteed by the manufacturer of the instrument (CV for WBC ≤ 3%). The manufacturer did not supply criteria for repeatability of the HPC value measurements; however, our data (CV = 11.4%) was consistent with reports from the literature [1]. The CV of the HPC count may have been influenced by the low absolute HPC numbers in certain apheresis products (error on small numbers of events).

**Figure 6:**
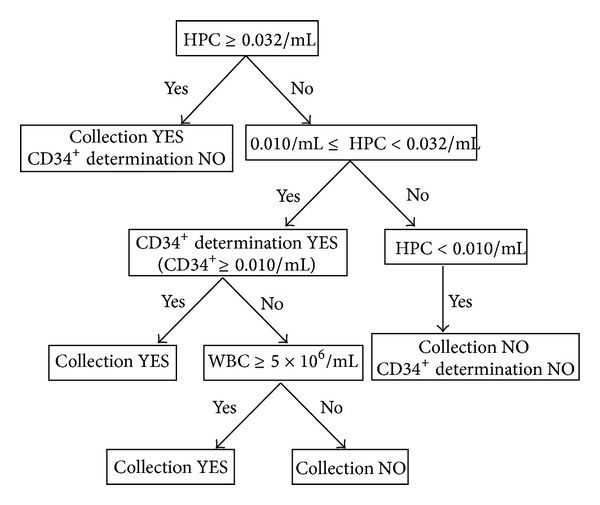
Protocol for the investigation of HPC in the peripheral blood. The results of our study showed that if the number of HPC cells was higher than 32/*μ*L, the quality of the PBSC concentrate was good (i.e., the yield of CD34^+^ cells was sufficient); because the results of HPC enumeration are reliable, there is no need to measure the number of CD34^+^ cells. In situations when the HPC number is between 10 and 32/*μ*L, we recommend an additional CD34^+^ cell count to precisely determine the time suitable for the initiation of PBSC collection (because while the HPC numbers and CD34^+^ expression in peripheral blood correlated very well, the correlation, however, was found to be weak between the HPC numbers and the yield). If the HPC number is lower than 10/*μ*L, there is no hope of a sufficient yield and it is not necessary to verify this situation by CD34^+^ cell enumeration; the collection in this case is not initiated.

Although the HPC method, in the peripheral blood, demonstrated a strong correlation to the reference flow cytometry CD34^+^ method (*R* = 0.604), differences were observed. The quantity of HPCs were an average of 3 times higher than the CD34^+^ cell values measured using the standard flow cytometry method (Table 1). The CD34^+^ cell population is not homogenous and different analysis methods can record different subtypes of cells. Nevertheless, the observation that the results, reported in literature for the same ISHAGE protocol, were different should not be ignored [22]. Measuring HPCs is a fast and economical method that can be used in a complementary fashion to determine the optimal timing for PBSC collection. Based on our experience, we implemented the results into the schema of our investigation protocol for PBSC collection (Figure 6).

Our results demonstrated that in the case of peripheral blood samples containing very low PBSC counts (HPC < 0.010 cells/mL), it was sufficient to determine only the HPC number without a parallel examination of the CD34^+^ cells. In this scenario, it would not be advisable to initiate the collection because the resulting yield of PBSCs would be very low and similar recommendations have been reported in the literature [23–25]. However, the HPC examination is sufficient for quick decisions. When the number of HPCs in the peripheral blood did not reach 0.010 cells/mL, the sensitivity and the NPV of the HPC method were high (sensitivity 93.6%; NPV 93.9%). When the HPC count is between 0.010–0.032 cells/mL, we recommend parallel determination of the CD34^+^ cell count using flow cytometry (again, our results are consistent with published reports [1, 23]) to correctly time the initiation of the PBSC harvest. In this scenario, even though the correlation analysis between HPC and the reference CD34^+^ cell number demonstrated a very good correlation in the peripheral blood, the correlation was weak only in the apheresis product (*R* = 0.464). We observed cases with a HPC count between 10 and 32 cells/mL in which the determined minimal yield (2 × 10^6^ CD34^+^ cells/kg in the recipient) was not reached. In our group, we performed 184 aphereses in which the yield was less than 2 × 10^6^ cells/kg in one day. We had 49 cases (26.6%) from this group with a HPC count between 0.010 and 0.032 cells/mL of which 26 cases (53%) exhibited a CD34^+^ cell count less than 0.010 cells/mL. Therefore, in the case of HPC count between 0.010 and 0.032 cells/mL, it is necessary to determine the HPC and CD34^+^ cell counts in parallel for a more accurate prediction.

An HPC count >0.032 cells/mL was sufficient in all cases for the initiation of the PBSC collection, even without a parallel examination of the CD34^+^ cells using the standard method and the quality of the PBSC concentrate was always sufficient. Our data indicated that the classic CD34^+^ determination method was not necessary and the decision for yield initiation can be made based on the HPC count, which is a faster and cheaper method. Importantly, a number of authors recommend a higher cut-off value for the HPC method (i.e., 0.045–0.050 HPC/mL) for the initiation of collection [2, 26, 27].

It is interesting that the HPC method correlated well with the standard CD34^+^ cell determination in the peripheral blood but the HPC count agreed only weakly with the reference method in the collection product (*R* = 0.464) and similar results have been reported by Vogel et al. [28]. Our group of patients was relatively small and it is possible that the correlation index could increase if more patients are used.

The setting of the CD34^+^ cells' cut-off value, appropriate for starting the collection of PBSC, was based on the literature as well as on our experience. Letestu et al., 2007 [1] started the separation when the value of 0.010–0.020 CD34^+^ cells/mL was reached while the number of WBC was at least 5 × 10^6^/mL. In our study, however, we had patients with 0.010 CD34^+^ cell/mL and less than 5 × 10^6^ WBC/mL (Table 2). Therefore, we chose the concentration 0.020 CD34^+^ cells/mL as the cut-off value for the ROC curve calculation. This is the higher recommended value, in accordance with the choice of other authors [1]. This evaluation does not conflict with our other results or with our examination protocol. Moreover, in the case of the so-called “gray zone,” when the number of HPC varies between 0.010 and 0.032/mL, we also performed a parallel analysis of the CD34^+^ cells with regard to the total number of WBC.

Hematopoietic stem cells (HSCs) and progenitors are maintained and are replenished in the blood at a steady state after PBSC or bone marrow transplantations. The HSCs themselves provide long-term hematopoietic repopulation of all blood cell lineages [8, 29, 30]. Progenitor cells are more restricted in their ability to proliferate and in their capacity to generate multiple cell lineages [17]. The CD34^+^ cell surface antigen is expressed in human hematopoietic stem cells and progenitors and is widely used to characterise these cells [31]. However, CD34^+^ cells are heterogeneous and recent evidence suggests that not all HSCs or progenitors always express the CD34 marker [17, 18]. A promising complementary strategy for identifying and studying hematopoietic stem cells and progenitors is the expression of intracellular enzymes that may be important during development, such as aldehyde dehydrogenase. ALDH may play an important role in retinoid metabolism and the enzyme appears to be expressed at moderately high levels in primitive hematopoietic cells in a number of species [32]. Collectively, these observations suggest that the expressions of ALDH and CD34 can be used to distinguish the developmental stages of human hematopoiesis. The most primitive hematopoietic cells express ALDH and CD34 and CD34 expression and ALDH expression are maintained throughout early myeloid differentiation. However, during lymphoid differentiation, ALDH expression apparently diminishes before the loss of CD34 expression. Therefore, the ALDH expression could be related to cell vitality and from this perspective may be an interesting way to evaluate the quality of the PBSC concentrates [18]. These observations compelled us to investigate the clinical significance of ALDH for optimal timing of the initiation of the PBSC harvest. Of course, it was not expected that the ALDH method would be faster and cheaper than the HPC enumeration using the flow cytometry method.

The data from the ALDH, CD133^+^ cell, and CD34^+^/CD133^+^ cell determinations were strongly consistent with the reference method for both the peripheral blood and the leukapheresis product. Therefore, these three alternative methods for the determination of the PBSC number in the peripheral blood or the collected concentrate samples could be used successfully to replace the existing CD34^+^ method. However, the three alternative methods did not prove to be less time-consuming or less costly than the original method; therefore, the methods do not exhibit a clear advantage over the standard method in clinical practice. Nevertheless, we assume that the enzymatic ALDH method will contribute to the investigation of graft vitality after graft thawing and before PBSC transplantation, mainly because it provides a significantly faster result (2 hours) compared, for example, with the cultivation of CFU-GM (colony-forming unit granulocyte-monocyte) progenitors, which is considered a reliable test but takes approximately 14 days [11].

## 5. Conclusion

In summary, four alternative methods to supplement or replace the standard flow cytometry determination of CD34^+^ cells were investigated; however, only the HPC method exhibited a practical benefit for optimal apheresis timing. The HPC method is fast, cheap, and easy to perform and has a very short response time. If necessary, the method can be performed more than once a day along with common blood count tests. Although we have been using the HPC method in our daily clinical practice for more than 3 years and recommend the method, it cannot completely replace the reference CD34^+^ method.

The main advantage of the HPC method is the quick result, which makes the method suitable for use in the therapeutic process using the specific algorithm.

## Figures and Tables

**Table 1:** Characteristics of the patients and general data concerning leukaphereses.

Summary statistics; general data	
Total number of patients (M/F)	84 (47/37)
Age (mean; median; range)	53; 58; 19–67 years
Dg.: MM, HL + NHL, AL, solid tumor/donors	49, 24, 3, 2/6
Dose of growth factor	5–10 *μ*g/kg/day
Start of PBSC collection (harvest)	6th–12th day
Duration of leukapheresis (mean; range)	244; 125–286 minutes
Number of leukaphereses (total number; mean; range)	266; 3; 1–6 days
PBSC yield (mean; range)	5.9; 0.1–21.3 × 10^6^ CD34^+^ cells/kg

M = male; F = female; MM = multiple myeloma; HL = Hodgkin lymphoma; NHL = non-Hodgkin lymphoma; PBSC = peripheral blood stem cells.

**Table 2:** Summary statistics of the laboratory results—peripheral blood.

Summary statistics; peripheral blood				
	Number of examinations	Range	Median	Mean
WBC × 10^6^/mL	266	2.90–71.64	22.03	24.41
HPC/mL	264	0–0.892	0.041	0.076
ALDH/mL	145	0–0.279	0.020	0.037
CD133^+^/mL	178	0–0.218	0.013	0.020
CD34^+^/CD133^+^/mL	178	0–0.204	0.009	0.017
CD34^+^/mL	265	0–0.255	0.015	0.025

WBC = white blood cells; HPC = hematopoietic progenitor cells; ALDH = aldehyde dehydrogenase.

**Table 3:** Summary statistics of the laboratory results—apheresis product.

Summary statistics; apheresis product				
	Number of examinations	Range	Median	Mean
WBC × 10^6^/mL	266	48.49–492.19	198.22	206.16
HPC/mL	264	0–6.691	0.692	1.247
ALDH/mL	142	0–8.723	0.688	1.192
CD133^+^/mL	182	0–4.347	0.378	0.708
CD34^+^/CD133^+^/mL	182	0–4.210	0.366	0.692
CD34^+^/mL	265	0–5.112	0.481	0.890

WBC = white blood cells; HPC = hematopoietic progenitor cells; ALDH = aldehyde dehydrogenase.
